# The effect of sandblasting versus acid etching on the surface roughness and biaxial flexural strength of CAD/CAM resin-matrix ceramics (In vitro study)

**DOI:** 10.1186/s12903-023-02883-6

**Published:** 2023-03-24

**Authors:** Heba A. Muhammed, Elsayed M. Mahmoud, Amal E. Fahmy, Dina M. Nasr

**Affiliations:** 1 Conservative Dentistry Department, Faculty of Dentistry, Alexandria University, Alexandria, Egypt; 2 Dental Biomaterials Department, Faculty of Dentistry, Alexandria University, Alexandria, Egypt

**Keywords:** Resin nano-ceramic, Flexible hybrid ceramic, Surface roughness, Flexural strength

## Abstract

**Background:**

CAD/CAM resin matrix ceramics are one of the materials used in dental offices. The long-term success of the restoration depends on the bond strength of the restoration to the tooth and other materials; thus, surface treatment of the restoration is necessary to achieve this. But such treatment may affect the restoration strength. The purpose of this study is to assess the impact of various surface treatments on the surface roughness (Ra) and the biaxial flexural strength of two CAD-CAM resin-matrix ceramics.

**Methods:**

Thirty-six-disc-shaped specimens, each measuring 1.2 mm in thickness and 12 mm in diameter, were machined from two resin-matrix ceramic blocks (Lava Ultimate and Cerasmart) (*n* = 18). Based on the surface treatments, each material was divided into 3 groups: control (no treatment), 50-μm Al_2_O_3_ sandblasting, or 9% hydrofluoric acid etching (*n* = 6). The surface roughness (Ra) was evaluated by the 3D laser scanning microscope. Then, specimens were aged by thermal cycling (5000 cycles) and tested for biaxial flexural strength using a universal testing machine at a crosshead speed of 1.0 mm/min.

**Results:**

No significant differences in flexural strength or Ra were found for Lava Ultimate among the surface treatment groups. For Cerasmart, only the sandblasting group showed significantly higher Ra values than the control group. Also, the Ra values for the sandblasting group were significantly higher than those for the acid etching group. The flexural strengths of the sandblasting and acid etching groups for Cerasmart were statistically similar, and both were significantly lower than the control group.

**Conclusions:**

Although all of the applied surface conditioning techniques improved Ra, they had a negative impact on the flexural strength of resin-matrix ceramics. Thus, clinicians should utilize the appropriate surface treatment techniques, taking into account their effects on the surface roughness and mechanical properties of resin-matrix ceramics.

## Background

In recent years, there has been a noticeable rise in the usage of tooth-colored indirect dental restorations made with computer-aided design and computer-aided manufacturing (CAD/CAM) technology [1]. To obtain the necessary qualities for restorations, including excellent aesthetics and function, a variety of CAD/CAM materials have been developed [2, 3].

Two materials that are frequently used in aesthetic dentistry are ceramics and composite resins [4]. Ceramics offer excellent optical and mechanical properties, chemical stability, as well as biocompatibility [5]. Unfortunately, they have disadvantages such as brittleness and abrasion of the opposing dentition [6]. Contrarily, composites don’t have these drawbacks but they have poor mechanical qualities, low biocompatibility, and surface discoloration, which motivated the introduction of resin matrix ceramic (RMC) materials [3, 6, 7]. These materials blend the best qualities of ceramic and composite materials and mitigate their drawbacks [8].

Unlike conventional resin composites, RMCs permit an increase in the filler volume capacity [9]. These materials are industrially polymerized at high pressures and temperatures, which is supposed to improve the material’s mechanical and physical properties [10, 11]. As opposed to the 50–60% conversion rate of direct resin composites, these materials have up to a 95% conversion rate, which results in improved biocompatibility [12]. These materials show less abrasion of antagonist dentition, and brittleness than ceramics [2, 13]. In addition, they offer more precise and smoother milled margins [14]. Its elasticity is similar to that of dentin [9]. The majority of them have been recommended for veneers, inlays, onlays, and crowns [15].

Resin nanoceramic (RNC, e.g., Lava Ultimate) and flexible hybrid ceramic (FHC, e.g., Cerasmart) are two types of CAD/CAM RMC blocks [16]. Lava Ultimate is the first RNC material [17]. It is composed of 80% nano zirconia–silica particles (69% SiO_2_ and 31% ZrO_2_) and 20% resin matrix [7]. On the other hand, Cerasmart has a flexible nano-ceramic matrix that contains homogenously dispersed 71% silica and barium glass nanoparticles [18].

The restoration’s clinical performance is significantly impacted by a strong bond between the restorative material and the cement [5]. It is maintained by chemical adhesion and mechanical interlocking [19]. There are several surface treatment strategies, and the best one depends on the material’s microstructure, and composition [20]. Mechanical surface treatments produce micro-retentive grooves and improve the wettability and roughness of the internal restoration surface [5, 19]. The prevalent mechanical surface treatments are acid etching and air abrasion [13].

Hydrofluoric acid etching induces microporosities by removing the glass matrix from the material substrate [20]. Etching causes topographic changes based on the etchant concentration, application time, and composition of the restorative material [21]. Sandblasting with aluminum oxide (Al_2_O_3_) is used to clean and improve a material’s surface roughness [22]. Consequently, enhancing surface activity and adhesion area [20]. The indentation pattern of the sandblasted material surface varied according to the particle size, propulsion pressure, and sandblasting duration [21]. The higher pressure of sandblasting (3 bar) can damage the material surface and reduce the strength of the restoration [22]. Thus, reduced pressure should be used [19]. Surface roughness explains the impact of pre-treatment techniques on the surface morphology of the material [1, 13]. Surface roughness is often expressed as (Ra), which represents the average of the microscopic peaks and valleys of the measured surface [23].

Thermal stresses occur in RMCs due to their diverse composition and the various coefficients of thermal expansion of their constituents. Aging that occurs in RMCs can reduce their clinical lifespan. Thermal cycling (TC) has been described as a methodology for promoting the materials’ mechanical characteristics degradation [15].

No single property can indicate a material’s clinical longevity, but flexural strength helps in providing an understanding of a material’s dynamic behavior at simulated occlusal stress [6]. Since CAD/CAM restorations’ bulk fracture is the primary reason for failure [11]. Flexural forces are applied to imitate clinical circumstances where materials must endure flexing, particularly in the posterior area that is exposed to multiaxial loading [11, 15]. A biaxial flexural strength (BFS) test was used to examine the flexural characteristics of samples with size limitations. BFS tests were done with a range of loading configurations, including piston-on-three-ball, ring-on-ring, and ball-on-ring (BOR). BOR is a simple testing device that provides more precisely controlled biaxial stress [11].

Due to the lack of agreement on the optimal surface treatment for RMCs and how surface treatment will affect the restorative material’s mechanical properties. The purpose of this study was to assess the impact of various surface treatments on the surface roughness of two different RMCs. Another aim of this study was to evaluate how the various surface treatments affected the materials’ BFS following thermocycling aging. The first null hypothesis of this study stated that there was no difference in the surface roughness of the RMCs among the various surface treatments. The second null hypothesis was that the BFS of the RMCs would not be impacted by the surface treatment technique.

## Methods

### Sample size estimation

The estimated sample size is based on the study’s 95% confidence level and 80% power. Based on previous studies, the mean ± SD surface roughness when surfaces were not treated = 0.27 ± 0.03, with air particle abrasion = 1.52 ± 0.16, and when treated with 9% hydrofluoric (HF) acid and silane, the mean ± SD = 0.39 ± 0.05. To ensure adequate power across all comparisons, the sample size was calculated based on the differences between no surface treatment and 9% HF acid and silane application [24]. The minimal required sample size was found to be 4 specimens per group, increased to 6 to make up for laboratory errors. The total sample size = the number of groups × number per group = 6 × 6 = 36. The sample size was based on Rosner’s method [25], as calculated by GPower 3.1 [26].

### Specimen fabrication

The brands, manufacturers, and chemical compositions of the materials used in this study are presented in Table 1. Thirty-six discs in total were milled from Lava Ultimate (A2 LT 14L) and Cerasmart blocks (A2 LT 14) with thicknesses of 1.2 mm and diameters of 12 mm [6]. To get rid of any surface contaminants, each disc was ultrasonically cleaned in three alternate solutions for three minutes each (distilled water, ethanol [95%], and distilled water) [27].

**Table 1:** Composition of the materials used in the study

Manufacturer	Composition	Classification	Material
3 M ESPE, St. Paul, MN, USA	**Organic:** 20 wt.% resin matrix (Bis-GMA, UDMA, Bis-EMA, TEGDMA) **Fillers:** SiO_2_ (20 nm), ZrO_2_ (4–11 nm), ZrO_2_/SiO_2_ nanoclusters (0.6–10 nm) **Filler content:** 80 wt.% nanoceramic	Resin nano ceramic	Lava Ultimate CAD/CAM blocks
GC Corporation, Tokyo, Japan	**Organic:**29 wt.% resin matrix (UDMA, DMA, Bis-MEPP**)** **Fillers:** barium glass (300 nm), SiO_2_ (20 nm) **Filler content:** 71 wt.% nanoparticles	Flexible nano ceramic	Cerasmart CAD/CAM blocks
Ultradent; South Jordan, UT, USA	Hydrofluoric acid 9% buffered	Hydrofluoric acid gel	Ultradent Porcelain Etch
Renfert GmbH, Hilzingen, Germany	99.5% Al_2_O_3_ (50 μm), SiO_2_ < 0.06%	Grit blasting particles	Cobra 50-μm Al_2_O_3_ powder

*Abbreviations: UDMA* urethane dimethacrylate, *Bis-GMA* bisphenol A-glycidyl methacrylate, *Bis-EMA* bisphenol A-ethyl methacrylate; *TEGDMA* triethylene glycol dimethacrylate, *Bis-MEPP* bismethacryloxyethoxydiphenylpropane, *DMA* dimethacrylate

### Grouping of the specimens

The discs were divided into two categories based on the material utilized. Group I (*n* = 18); Lava Ultimate (LU); Group II (*n* = 18); Cerasmart (CS). Then, each group was divided into three subgroups based on the used surface treatment (*n* = 6).

- *Control group (C):* disc surfaces left untreated.
- *Sandblasting group (SB):* discs were sandblasted with 50-μm of Al_2_O_3_ particles at 2 bar pressure for 10 s at a 10-mm distance with a circular motion [14, 28]. A permanent marker was used to paint the targeted surface, and when the ink was entirely erased, the entire surface was treated [21].
- *Hydrofluoric acid group (HF):* discs were etched with 9% HF acid for 60 s, followed by a 2-min rinse and drying [5, 12].

After all surface treatments, the discs were cleaned in a distilled water ultrasonic bath for 5 min [29].

### Surface roughness analysis

The surface roughness value (Ra) of all the specimens was measured using a 3D laser scanning microscope (Keyence VK-X100, Osaka, Japan). All measurements were done with a 20-x magnification [28]. On each sample, three different locations were measured. By averaging the three Ra values for each specimen, the mean Ra (μm) was calculated. Also, 3D images of the measured surfaces were recorded for a qualitative evaluation of surface roughness. The obtained images were interpreted using graphics and a color scale.

### Aging of the specimens

After the surface roughness measurements, all the specimens were aged using a thermocycling machine. A total of 5,000 cycles at 5 and 55 °C, corresponding to about six months of clinical use, were done [4]. The dwell time was set at 30 s, while the transition time was set at 3 s.

### Biaxial flexural strength test

The biaxial flexural strength test of all the samples was carried out by a BOR assembly [11]. Each disc was put on a specially designed jig with 1-mm circumferential support [15]. A ball-end plunger with a 3.2 mm diameter was used to apply a gradually increasing load to the discs until they fractured. The test was conducted using a universal testing machine with a cross-head speed of 1.0 mm/min. The highest load was noted in N, and the BFS was determined in MPa using the following equation [11].

$${\upsigma }_{BOR}=\frac{3P\left(1+\upsilon \right)}{{4\pi t}^{2}}\left[1+2\mathrm{ln}\frac{a}{b}+\frac{\left(1-\upsilon \right)}{\left(1+\upsilon \right)}\left(1-\frac{\mathrm{b}}{2{a}^{2}}\right)\frac{{a}^{2}}{{r}^{2}}\right]$$

where P is the maximum load at failure, ʋ is Poisson’s ratio (0.302 for LU, and 0.306 for CS) [11], a is the supporting circle radius (5 mm), r is the disc radius, t is the disc thickness, and b is the radius of the region of uniform loading at the center.

### Surface topography examination

Representative specimens were selected from each studied group. Then samples were adhered to metallic stubs with double-sided adhesive tape and finely coated with gold using an ion sputtering device (JFC-1100, JEOL, Tokyo, Japan). Micrographs of specimens of each material were taken at 300 × and 4000 × magnifications by a scanning electron microscope (SEM) (JSM-IT200, JEOL, Tokyo, Japan) working at an accelerating voltage of 15 kV to evaluate surface topography.

### Statistical analysis

The normality was checked using the Shapiro–Wilk test, and Boxplot, and it was found to be not normally distributed. The Kruskal–Wallis test was applied to assess the difference between groups, followed by Dunn’s post hoc test with Bonferroni Correction. The significance level was set at a p-value of 0.05. The data were analyzed using IBM SPSS version 23.

## Results

### Surface roughness (Ra)

**Fig. 1:**
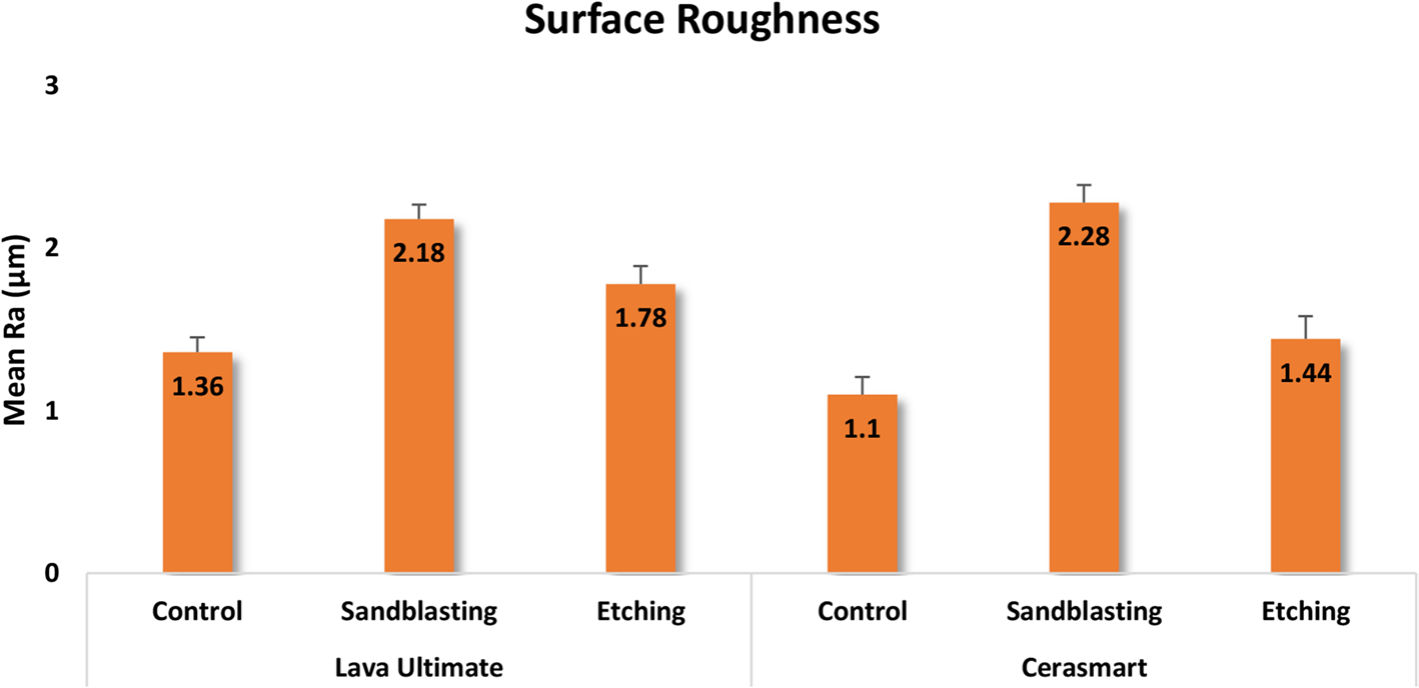
Bar chart graph showing the mean surface roughness (Ra) values in (μm) among the study groups

Range (minimum and maximum), mean, standard deviation, and median were used to describe quantitative data of the investigated materials’ surface roughness after various surface treatments, as shown in Table 2. The results of the Kruskal–Wallis test revealed that surface roughness was significantly affected by surface treatments (*p* < 0.0001). The order of Ra values according to the tested surface treatment was SB > HF > C. The mean surface roughness values for the study groups are shown in the bar chart graph (Fig. 1).

**Table 2:** Comparison of surface roughness (Ra) in micrometers among the study groups

	**Lava Ultimate**			**Cerasmart**		
	Control (*n* = 6)	Sandblasting (*n* = 6)	Etching (*n* = 6)	Control (*n* = 6)	Sandblasting (*n* = 6)	Etching (*n* = 6)
Mean (SD)	1.36 (0.09)	2.18 (0.09)	1.78 (0.11)	1.10 (0.11)	2.28 (0.11)	1.44 (0.14)
Median	1.38	2.18	1.81	1.12	2.27	1.41
Min – Max	1.22 – 1.49	2.05 – 2.32	1.58 – 1.90	0.91 – 1.22	2.18 – 2.39	1.24 – 1.60
Test (*P* value)	32.167 (< 0.0001*)					

^*^Statistically significant at *p*-value ≤ 0.05

The results of the post hoc test for the Pairwise comparison of surface roughness amongst the study groups are shown in Table 3. The results showed that the following pairs were not significantly different (*p* = 1.00): LU(C) vs. CS(C), LU(SB) vs. CS(SB), and LU(HF) vs. CS(HF). Regarding LU, there was no significant difference between the C and the SB groups (*p* = 0.063). In addition, no significant difference was obtained between C and HF groups or between the SB and HF groups (*p* = 1.00). For CS, a significant difference was detected between the C and SB groups (*p* = 0.0001) and between the SB and HF groups (*p* = 0.040). While the C and HF groups were not significantly different (*p* = 1.00).

**Table 3:** Pairwise comparison between the study groups regarding surface roughness

Group	Compared to	*P* value
Lava Ultimate (Control)	Lava Ultimate (Sandblasting)	0.063
	Lava Ultimate (Etching)	1.00
	Cerasmart (Control)	1.00
	Cerasmart (Sandblasting)	0.012*
	Cerasmart (Etching)	1.00
Lava Ultimate (Sandblasting)	Lava Ultimate (Etching)	1.00
	Cerasmart (Control)	0.0001*
	Cerasmart (Sandblasting)	1.00
	Cerasmart (Etching)	0.183
Lava Ultimate (Etching)	Cerasmart (Control)	0.055
	Cerasmart (Sandblasting)	1.00
	Cerasmart (Etching)	1.00
Cerasmart (Control)	Cerasmart (Sandblasting)	0.0001*
	Cerasmart (Etching)	1.00
Cerasmart (Sandblasting)	Cerasmart (Etching)	0.040*

^*^Statistically significant at *p*-value ≤ 0.05

**Fig. 2:**
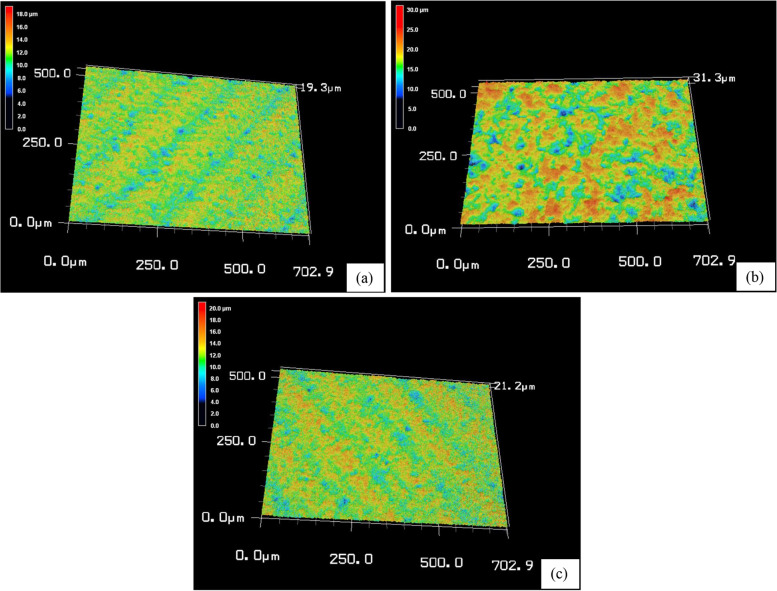
Representative 3D topographic maps of Lava Ultimate after different surface treatments: **a** control, **b** sandblasting, **c** HF acid etching

**Fig. 3:**
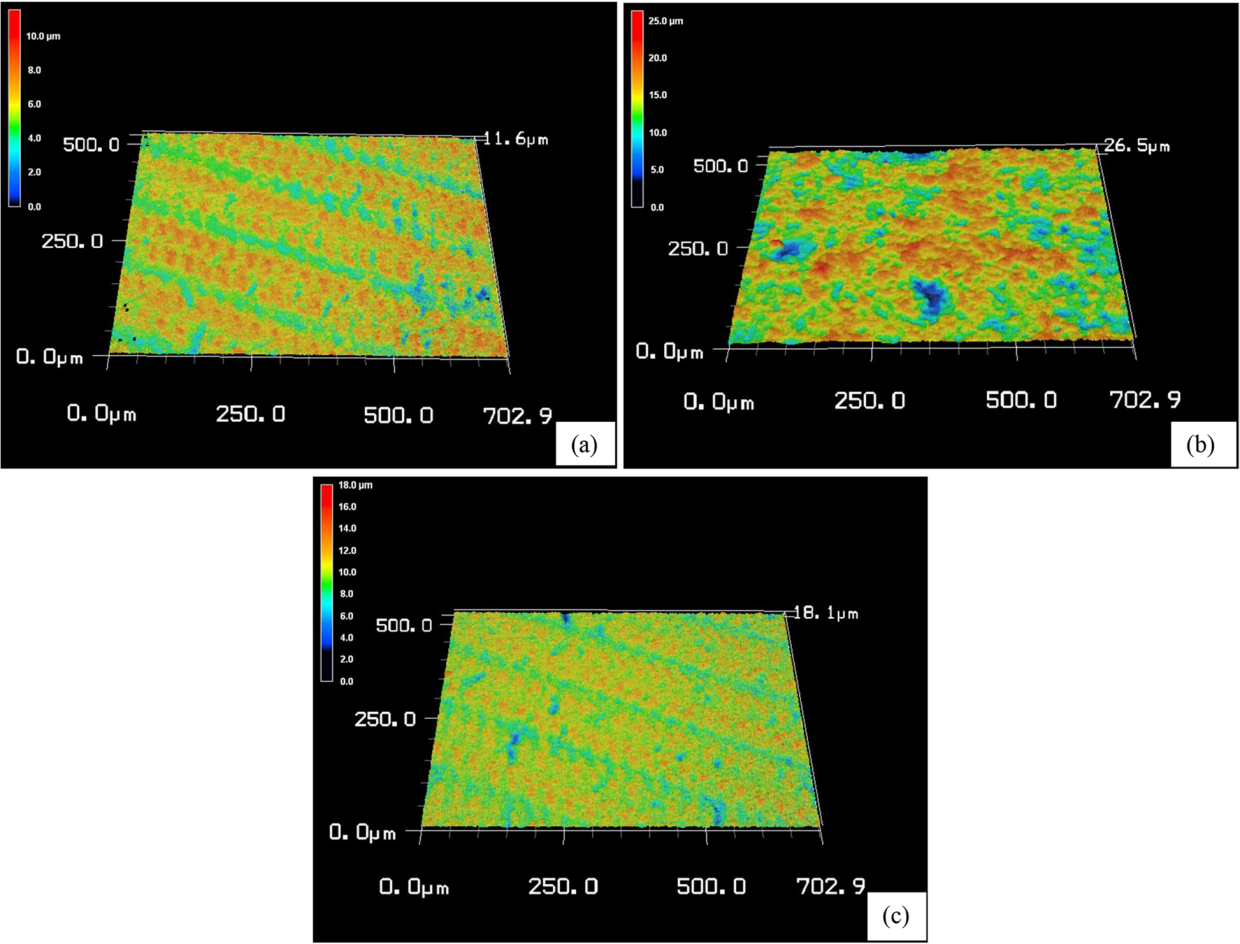
Representative 3D topographic maps of Cerasmart after different surface treatments: **a** control, **b** sandblasting, **c** HF acid etching

A qualitative analysis of the tested groups’ surface roughness data is displayed as 3D topographic maps in Figs. 2 and 3. Based on the surface treatment, various surface topographies were seen with distinct patterns of peaks and valleys.

### Biaxial flexural strength

**Fig. 4:**
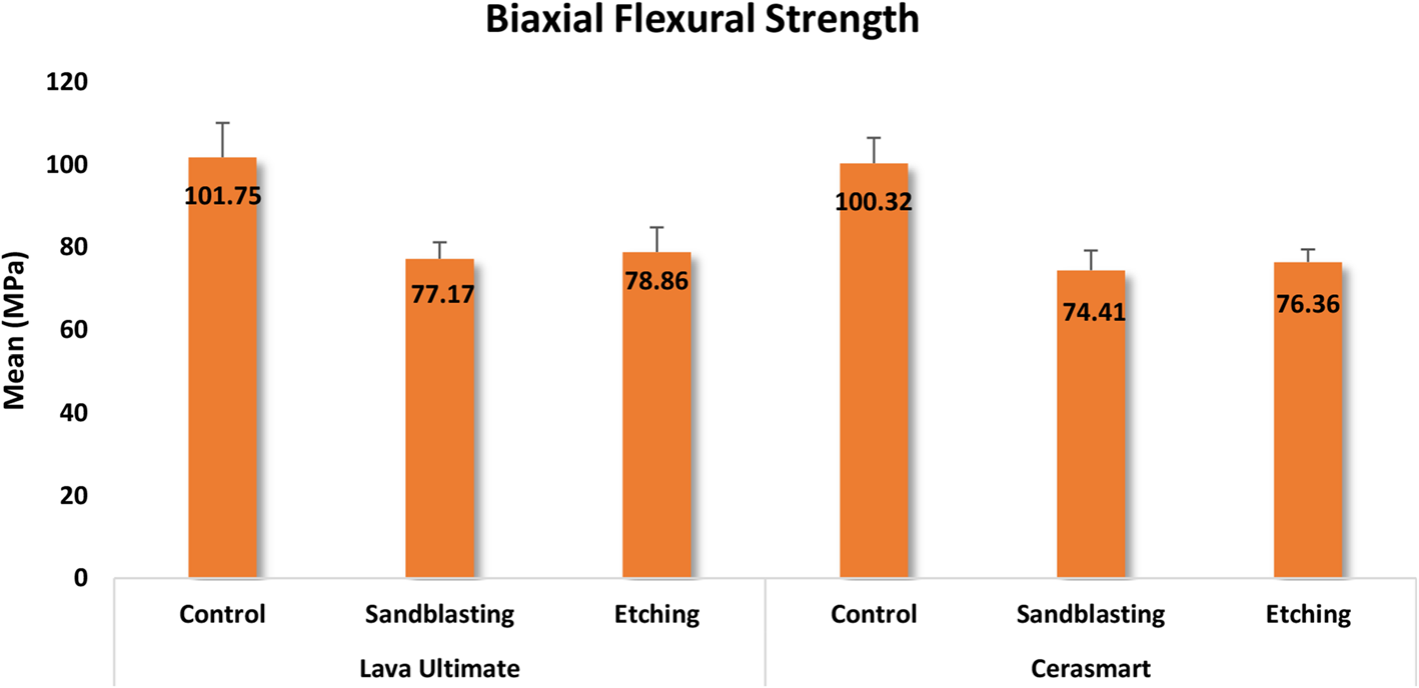
Bar chart graph showing the mean biaxial flexural strength (MPa) values among the study groups

Range (minimum and maximum), mean, standard deviation, and median were used to describe the quantitative data of the BFS for the investigated materials after various surface treatments, as shown in Table 4. The results of the Kruskal–Wallis revealed that BFS was significantly affected by surface treatments (*p* < 0.0001). The order of BFS values according to the tested surface treatment was C > HF > SB. The mean BFS values among the study groups are shown in the bar chart graph (Fig. 4).

**Table 4:** Comparison of biaxial flexural strength in MPa among the study groups

	**Lava Ultimate**			**Cerasmart**		
	Control (*n* = 6)	Sandblasting (*n* = 6)	Etching (*n* = 6)	Control (*n* = 6)	Sandblasting (*n* = 6)	Etching (*n* = 6)
Mean (SD)	101.75 (8.35)	77.17 (3.97)	78.86 (5.96)	100.32 (6.17)	74.41 (4.74)	76.36 (3.06)
Median	101.59	78.33	77.56	101.43	74.21	75.32
Min – Max	92.97 –112.33	71.31 – 81.28	71.91 – 88.5	92.79– 107.98	68.61 – 79.81	74.05 – 82.07
Test (*P* value)	24.345 (< 0.0001*)					

^*^Statistically significant at *p*-value ≤ 0.05

The results of the post hoc test for the Pairwise comparison of BFS between the study groups are shown in Table 5. The results showed that the following pairs were not significantly different (*p* = 1.00): LU(C) vs. CS(C), LU(SB) vs. CS(SB), and LU(HF) vs. CS(HF). Regarding LU, there was no significant difference between the C and the SB groups (*p* = 0.066), between the C and the HF groups (*p* = 0.128), or between the SB and the HF groups (*p* = 1.00). For the CS, a significant difference was detected between the C and SB groups (*p* = 0.010) and between the C and HF groups (*p* = 0.046). While it was found that there was no significant difference between the SB and the HF groups (*p* = 1.00).

**Table 5:** Pairwise comparison between the study groups regarding biaxial flexural strength

Group	Compared to	*P* value
Lava Ultimate (Control)	Lava Ultimate (Sandblasting)	0.066
	Lava Ultimate (Etching)	0.128
	Cerasmart (Control)	1.00
	Cerasmart (Sandblasting)	0.006*
	Cerasmart (Etching)	0.027*
Lava Ultimate (Sandblasting)	Lava Ultimate (Etching)	1.00
	Cerasmart (Control)	0.109
	Cerasmart (Sandblasting)	1.00
	Cerasmart (Etching)	1.00
Lava Ultimate (Etching)	Cerasmart (Control)	0.205
	Cerasmart (Sandblasting)	1.00
	Cerasmart (Etching)	1.00
Cerasmart (Control)	Cerasmart (Sandblasting)	0.010*
	Cerasmart (Etching)	0.046*
Cerasmart (Sandblasting)	Cerasmart (Etching)	1.00

^*^Statistically significant at *p*-value ≤ 0.05

### Surface Topography

**Fig. 5:**
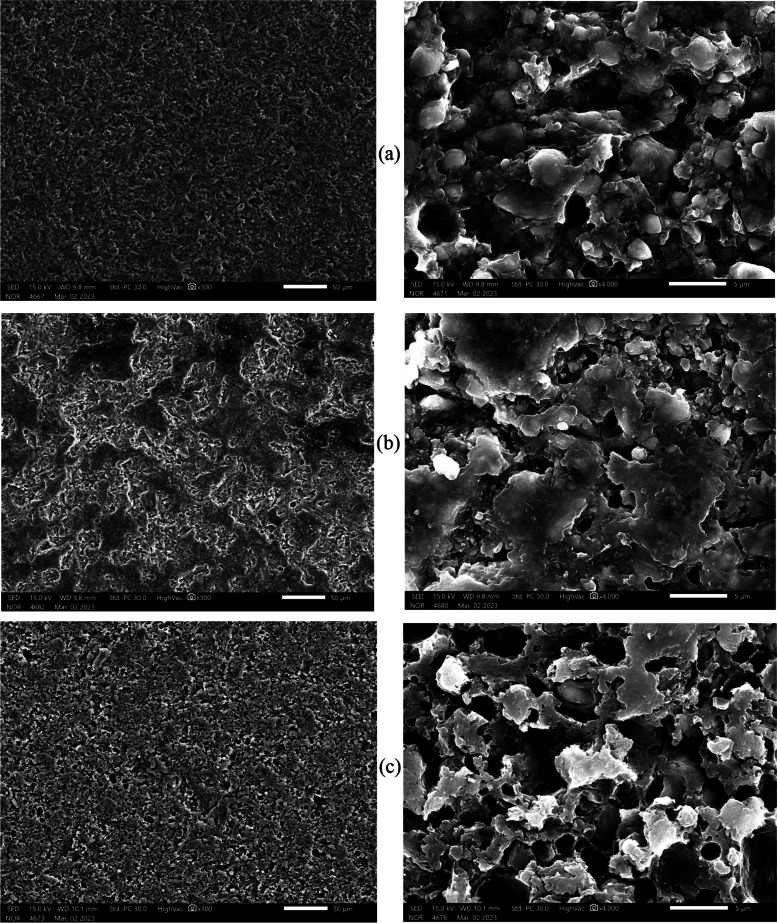
Representative SEM micrographs of Lava ultimate samples after different surface treatments: **a** control, **b** sandblasting, **c** HF acid etching. SEM images (left) at 300 × magnification and (right) at 4000 × magnification

**Fig. 6:**
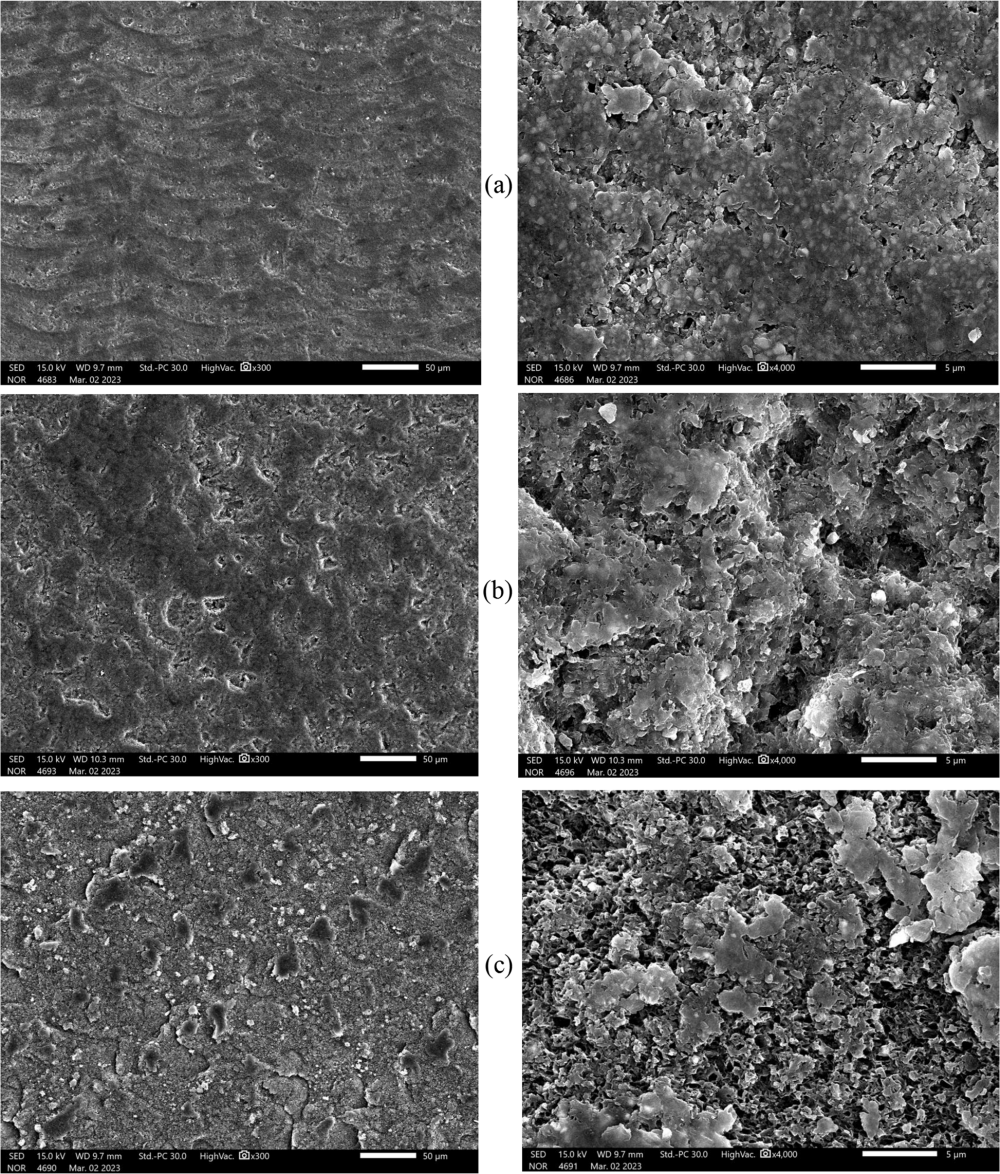
Representative SEM micrographs of Cerasmart samples after different surface treatments: **a** control, **b** sandblasting, **c** HF acid etching. SEM images (left) at 300 × magnification and (right) at 4000 × magnification

The SEM micrographs of the specimens from each tested group are shown in Figs. 5 and 6. Depending on the surface treatment and the RMC material, different surface topographies were observed. SEM micrograph analysis showed that the treated groups’ surface topography differed from that of the control group. The sandblasted specimens had surfaces that were morphologically roughened and had irregular craters of various sizes and shapes, as seen in (Figs. 5b–6b). While the specimens etched with HF acid showed a change in surface texture as well as irregular gaps and micropores as seen in (Figs. 5c–6c).

## Discussion

### Surface roughness

Ra is the most commonly used indicator to quantitatively describe the surface roughness [1, 30]. It offers practical and understandable value; therefore, it is still chosen for this study [6].

A first null hypothesis, which claimed that there was no difference between the different surface treatments in terms of the surface roughness of RMCs, was partially rejected. As CS had a statistically significant difference between the C and SB groups.

The LU (C) group is rougher than the CS (C) group as seen in (Figs. 2a–3a), even though all samples used the same CAD/CAM milling machine and technique. The variations between materials in the filler amount, and particle size might contribute to these results [3, 31]. In comparison to the 71 wt.% inorganic part of CS, LU had 80% filler content [2, 3, 31]. CS contains well-scattered nanofiller particles (10 to 30 nm) among medium-sized fillers (about 0.5 μm) without aggregated fillers [14, 31, 32]. Contrarily, LU includes larger filler clusters (about 10 μm) [31]. These clusters were made up of small spherical fillers (10 nm) and large core fillers (1–3 μm) [14]. Research has demonstrated that the amount and type of the organic matrix have an impact on the roughness values [2, 23]. Because materials’ molecular hardness values may be impacted by variations in the polymerization ratios of UDMA and Bis-GMA. However, it is challenging to compare different materials based on their organic content, because they are not explicitly defined due to company secrecy requirements [2]. Contrary to what we found, Egilmez et al. in 2018 showed that CS Ra values are significantly higher than those of LU [33]. This could be due to the various methodologies used.

In this study, a variety of pretreatments were applied to increase surface roughness while guaranteeing the materials’ surfaces sustain the least degree of damage. In 2018, Tekçe N studied the impact of SB times on the LU surface roughness and found that specimens sandblasted for 15 s featured micro-craters close to the surface. The samples that were sandblasted for 30 and 60 s revealed significant surface flaws like deep scratches, pitting, and grain pull-out. Additionally, several sand particles were detected embedded in the specimens’ surfaces after 60 s of SB [19]. In 2017, Yoshihara et al. examined the alterations in the microstructure that result from the SB RMC blocks. They observed that when the surface was blasted with 50- μm particles at 2 bars, the RMC’s surface roughness improved [14]. Thus, SB has been used in the current investigation with 50 μm Al_2_O_3_ particles at 2 bars with circular motion for 10 s.

The SB treatment in the current study presented both materials with a rougher surface than the HF treatment as seen in (Figs. 2b, c–3b, c). Similar findings were reported by Chuenjit P in 2021[20]. The CS group had higher surface roughness than the LU group, which coincides with the Alp et al. study in 2018 [34]. Since CS has lower flexural moduli and filler mass percentages than LU, it might be more susceptible to SB [20]. In addition, LU includes big clusters that are more difficult to dislodge from the surface of the material during SB [13]. Surface roughness and mechanical characteristics of materials are strongly correlated, according to research by Flury et al. in 2017 [35]. So, harder materials are expected to be less vulnerable to that type of surface treatment [29]. The results from Sismanoglu et al. in 2020 showed that LU SB samples had significantly higher surface roughness than HF samples [12].

Because HF acid is a fluorine-containing etchant that has been found to induce a roughened surface in the majority of acid-sensitive ceramics and polymers, it was chosen in the present study [13]. The etched surface changed into a hexafluoro-silicate compound, which may be rinsed off with water to be microporous [20].

In this investigation, Ra values were relatively higher in the CS and LU surfaces following HF than in the C group as seen in (Figs. 2a, c–3a, c). This is consistent with the Strasser et al. study in 2018 [1]. Papadopoulos et al. in 2020 concluded that HF had less impact on eroding the glassy phase of the material surface and produced only minute gaps and micropores [13]. In contrast to our investigation, CHUENJIT P in 2021 showed that there was an obvious alteration in the etched surfaces of CS based on the SEM data [20]. These results differ from those published by El-Damanhoury et al. in 2021, who stated that CS had a relatively smoother surface following HF treatment [10].

### Surface topography

SEM analysis revealed that different surface treatments produced varying surface topography and roughness. The SB treatment significantly roughened the RMC’s surface in comparison to the HF etching, as seen in the SEM micrograph (Figs. 5b, c–6b, c). The SEM micrograph showed that the roughness increased significantly after the HF etching and SB treatments in comparison to the control, as seen in (Figs. 5 and 6). The findings of the current study were in agreement with investigations by Chuenjit et al. in 2021 and Sismanoglu et al. in 2020 [12, 20]. Also, the SEM results match those of the surface roughness test by a 3D laser scanning microscope. The fillers’ size, shape, and distribution varied depending on the material, as shown by SEM inspection of the RMCs. In a prior investigation of resin composites, the size, distribution of filler, and flexural characteristics were found to be correlated [36]. This can be one of the factors causing the variations in flexural characteristics between the materials.

### Biaxial flexural strength

For a long time, material strengths were assessed by uniaxial strength tests using three- or four-point rectangular samples. Only a partial description of the load-bearing capacity may be given by such measures [6]. Therefore, the BFS test was selected for this study. The null hypothesis that there wouldn’t be any variation in the BFS of the CAD/CAM materials following the different surface treatments was partially rejected. Because CS’s BFS was significantly impacted by the surface treatment.

The matrix and filler within a material have inherent features that determine its mechanical properties [37]. So, the two RMC materials do not behave similarly [9]. LU has a higher concentration of inorganic fillers and coarser microstructure than CS, which has been linked to better FS and fracture deflection [15, 38, 39]. The findings of the current study were in agreement with investigations by Niem et al. in 2019 and Shafter et al. in 2017 [40, 41].

While the manufacturer of LU only recommends using SB, CS can be treated with HF as well [5]. In this investigation, all of the recommended surface treatments reduced the evaluated materials’ BFS. Yoshihara et al. in 2017 examined the impact of SB on the surface topography of LU and CS, and they concluded that it negatively affected the material surfaces. SB caused cracks in the resin matrix and debonding of the filler particles [14]. On the other hand, the resin matrix is partially destroyed by HF. Others claimed that the glass fillers within the resin’s matrix completely disintegrated when subjected to HF [5]. In line with the current investigation, in 2020 Porto et al. observed that there was no significance in LU following various surface treatments [29]. Also, significant differences were seen in CS after the different surface treatments, according to Kurtulmus-Yilmaz et al. in 2019 [5].

The FS of materials having polymers in their composition has been reported to degrade during TC [15, 29]. This might explain by water infiltration in the matrix, which softens and swells the network, reducing frictional forces between polymer chains and cause stress corrosion on the filler surface [37, 40]. In addition, the hydrolysis of the interfacial silane coupling agent between the fillers and the resin matrix [7]. According to Tsujimoto et al., TC (5:60 °C, 10,000 cycles) resulted in significantly lower FS values for LU and CS than simple water storage (24 h, 37 °C) [36]. The investigations by Egilmez et al. also demonstrated that, for CS and LU, TC (5:55 °C, 5000 cycles) caused a considerable decrease in FS when compared to no-treatment specimens [42].

A positive association between the BFS value and the fragment number was shown. The high-strain energy accumulated during loading is what causes the many fragmentations in the biaxial discs [11]. That indicates materials can withstand more mechanical stress and elastic deformation without breaking [37]. It was found that LU samples split into fewer pieces than CS samples. LU also demonstrated a failure pattern in which the sample broke without separating into pieces.

The maximum load at failure for LU and CS, respectively, was 142.51 N and 137.11N. These values were close to the dentine’s (109 ± 10 MPa) value [43]. The findings in this study support earlier publications, indicating that these materials are appropriate for thin veneers, inlays, and onlays. And is not recommended for young individuals or patients with bruxism. As these values were greater than the maximal occlusal forces of the anterior teeth but lower than the one-sided bite forces of the posterior area [17].

Regarding the study’s limitations, additional tests might be required using specimens milled in clinically anatomical forms. Also, the impact of loading and the different surface treatments, together with silane, primer, and cement, on the bond strength of these materials should be tested in future research. Additional research may be required to evaluate the biaxial flexural strength after adding more age cycles or combining thermomechanical and aging processes.

## Conclusion

It was concluded that in light of the current study’s limitations.

1. Sandblasting significantly increased surface roughness for Cerasmart material when compared to acid etching and control groups.
2. In comparison to the control group, both sandblasting and acid etching significantly reduced the biaxial flexural strength of Cerasmart, with sandblasting achieving the lowest value.
3. No surface treatments had a significant effect on the surface roughness or biaxial flexural strength of Lava Ultimate.
4. Particularly when the restoration is thin, clinicians should utilize the appropriate surface treatment techniques, taking into account their effects on the surface roughness and mechanical properties of resin-matrix ceramics.
5. Additionally, these materials are better used in anterior and premolar regions where forces of mastication are lower than in molar regions.

## Data Availability

The datasets used and/or analyzed during the current study are available from the corresponding author upon reasonable request.

## Abbreviations

CAD/CAM: Computer-aided design and computer-aided manufacturing
RMC: Resin matrix ceramic
RNC: Resin-nanoceramic
FHC: Flexible hybrid ceramic
TC: Thermal cycling
FS: Flexural strength
BFS: Biaxial flexural strength
BOR: Ball-on-ring
LU: Lava Ultimate
CS: Cerasmart
SB: Sandblasting
HF: Hydrofluoric
SEM: Scanning electron microscope
